# Unique Presentation of Adult Ileocecal Intussusception Unveiling a Rare Culprit: A Carcinoid Tumor

**DOI:** 10.7759/cureus.59308

**Published:** 2024-04-29

**Authors:** Christopher M Zammit, Margaret L Munz, Lindsay Tjiattas-Saleski, Joshua Knolhoff

**Affiliations:** 1 Surgery, Edward Via College of Osteopathic Medicine, Spartanburg, USA; 2 Emergency Medicine, Edward Via College of Osteopathic Medicine, Spartanburg, USA; 3 General Surgery, Atrium Health, Columbus, USA

**Keywords:** ileum, lymph node dissection, right-sided hemicolectomy, bowel obstruction, carcinoid syndrome, colorectal surgery, intussusception, carcinoid tumor

## Abstract

Intussusception is a prominent contributor to bowel obstruction, marked by the invagination of a proximal bowel section into a distal segment. Ileocecal intussusception occurs when a portion of the terminal ileum folds into the cecum. In adults, intussusception is infrequent compared to pediatric cases, and represents a minority of bowel obstructions. Structural lead points are more commonly observed in adult intussusception compared to pediatric cases where risk factors include infection, Meckel’s diverticulum, and intestinal polyps. Obstructions in adults are usually a result of benign or malignant neoplasms. In this particular case, a carcinoid tumor originating in the ileum acted as the structural lead point for intussusception. The patient underwent exploratory laparotomy resulting in a right hemicolectomy. This patient did not present with the classic triad of symptoms associated with carcinoid syndrome. In response to this particular case, a laparoscopic right-sided hemicolectomy with lymph node dissection was performed.

## Introduction

Intussusception is a condition characterized by the telescoping of the proximal section of the bowel into the distal segment, potentially leading to bowel obstruction [1-3]. Ileocecal intussusception refers to the invagination of a portion of the terminal ileum into the cecum. Adult cases account for 5% of all intussusception cases and represent 1% of all adult bowel obstructions [3-5]. Intussusception is more commonly observed in pediatric patients [6].

While many cases are idiopathic, structural lead points are more frequently encountered in adult cases as opposed to pediatric cases, which tend to be primarily idiopathic in nature [6]. Risk factors include infections, Meckel’s diverticulum, intestinal polyps, fibroids, endometriosis, ostomy tubes and drains, and anatomical anomalies [7]. Post-operative risk factors consist of adhesions, suture lines, and intestinal tubes [6]. In adults, structural obstructions typically result from benign or malignant neoplasms, with lipoma being the most common cause of small bowel intussusception (38%) [8,9]. Colonic intussusception is predominantly associated with malignant lesions (63%), particularly adenocarcinomas and lymphomas [8,9].

Accurate diagnosis of intussusception can be challenging, often remaining elusive until the condition progresses to obstructive symptoms. A definitive diagnosis is achievable through CT scan, which carries a sensitivity of 71.4-87.5% and is considered the gold standard of diagnosis [6]. The “target-sign”, “doughnut-sign”, and “trident-sign” are frequently employed to describe the presentation of intussusception on CT [6]. 

First reported over 100 years ago, carcinoid tumors most commonly occur in the lungs, bronchi, and gastrointestinal tract [10]. These tumors originate from neuroendocrine cells known as enterochromaffin cells of Kulchinsky, which play a crucial role in the regulation of gastrointestinal motility and secretion [11]. Histologically, carcinoid tumors manifest as nests of tumor cells separated by fibrous septae and comprise up to half of all small intestine neoplasms [11]. Carcinoid tumors are sometimes functional and can result in a rare condition known as carcinoid syndrome [12]. This syndrome is a result of hormone production by the tumor and symptoms include diarrhea, flushing, abdominal cramping, and right-sided cardiac valvular anomalies [11,12]. Carcinoid tumors rarely present with carcinoid syndrome, especially without extensive liver metastasis [12]. Functional carcinoid tumors resulting in hormonal symptoms are treated with octreotide, a somatostatin analog [11].

The patient described in this case did not present with symptoms typical of carcinoid syndrome. Treatment for small bowel intussusception with obstructive symptoms necessitates addressing the underlying cause. As in this case, when a carcinoid tumor is found to be the culprit, definitive treatment with surgical resection is appropriate as well as screening for metastasis and surveillance [8].

This case was previously presented at the American College of Osteopathic Surgeons Conference on September 22, 2023.

## Case presentation

A 72-year-old male presented to the emergency department with a chief complaint of abdominal pain. The patient reported sudden onset, diffuse, periumbilical abdominal pain with nausea and vomiting. He denied any chest pain or shortness of breath and was afebrile. The patient admitted to recent loose stools, cramping, and bloating, and denied any unintentional weight loss.

The patient’s medical history was significant for hypertension and hyperlipidemia. He also had a surgical history including various orthopedic procedures, a right inguinal hernia repair, hemorrhoidectomy, spinal fusion, and dental procedures. There was a maternal family history of cancer of unknown origin. The patient’s social history was positive for only social use of alcohol, and he denied any smoking or illicit drug use.

**Figure 1:**
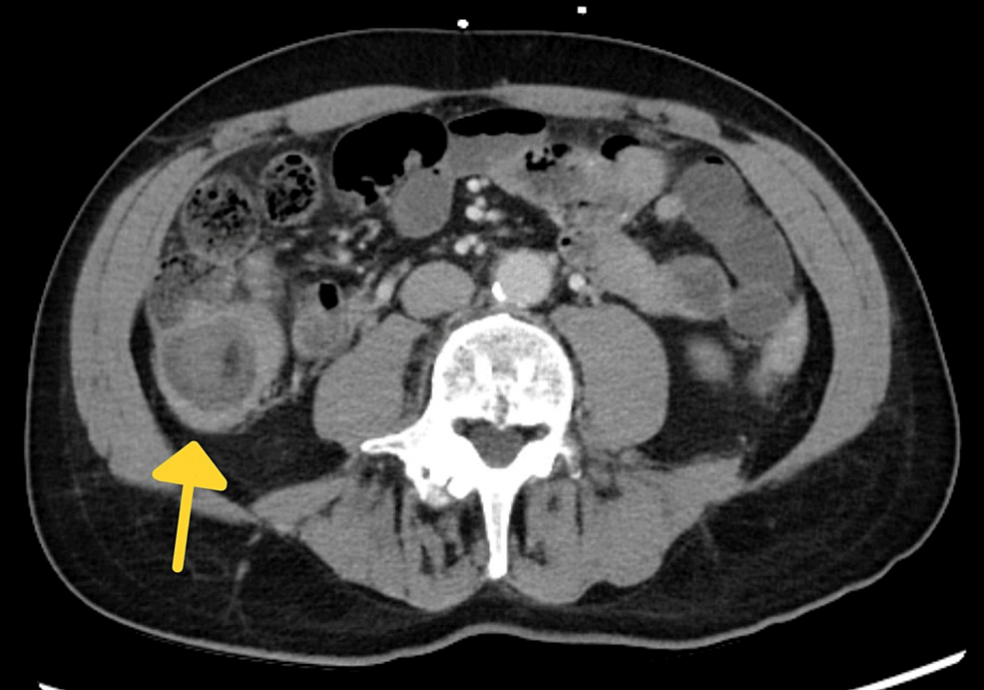
CT showing target sign of small bowel obstruction

**Figure 2:**
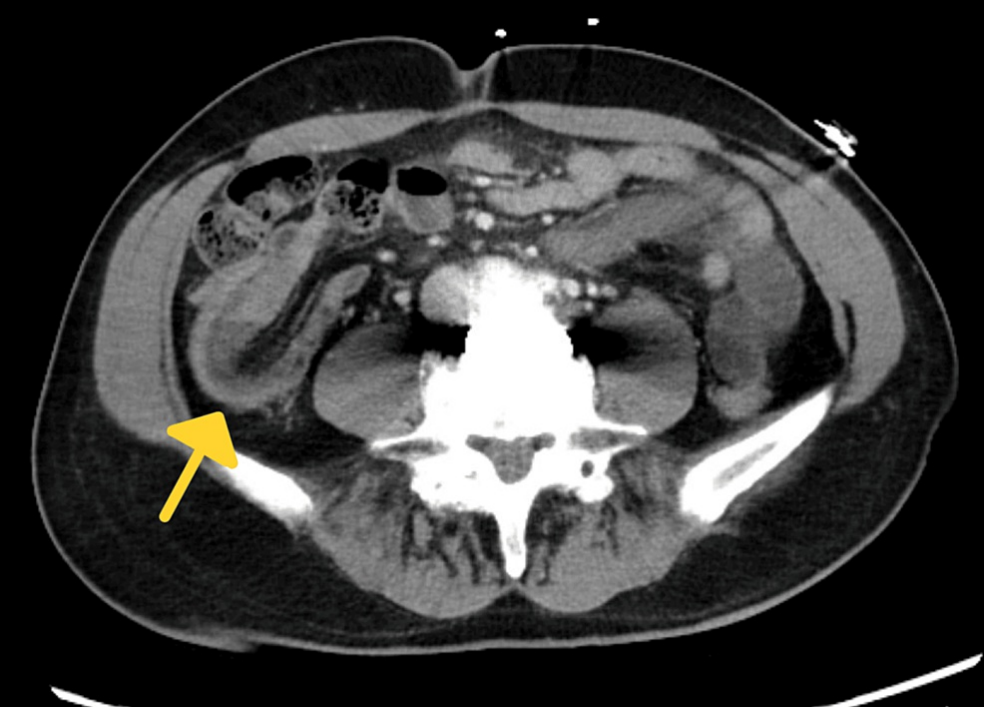
CT showing crescent sign with signs of small bowel obstruction

**Figure 3:**
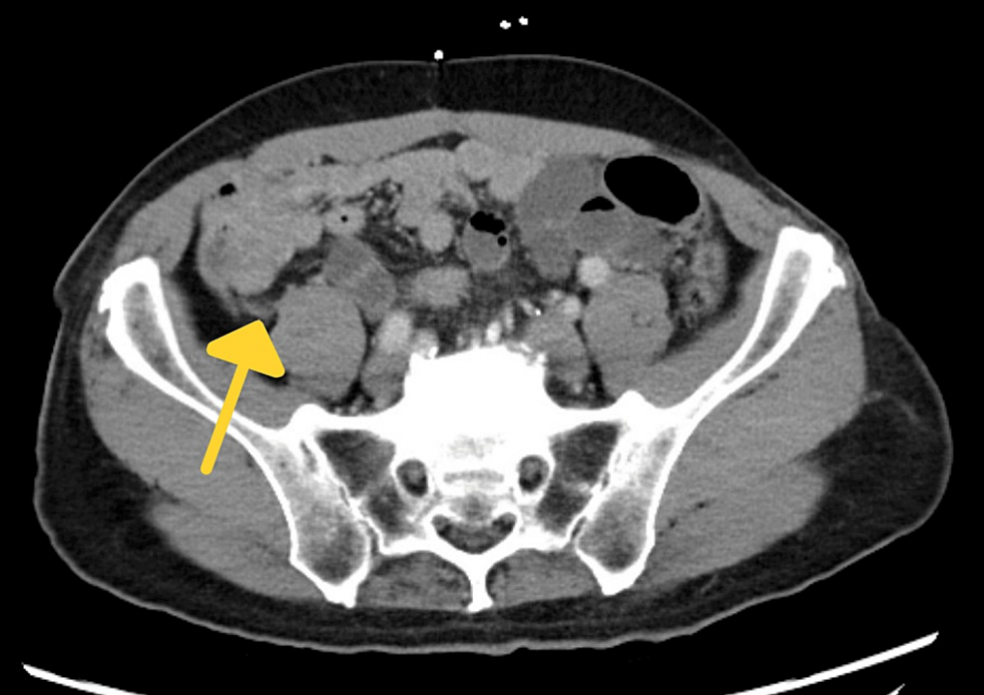
CT with arrow indicating appendix

On physical examination, the patient appeared to be an alert, well-nourished adult male in mild distress experiencing periumbilical abdominal discomfort. He had significant right lower quadrant tenderness without rebound or guarding. The abdomen was not notably distended and no organomegaly was appreciated upon palpation. The physical exam and labs were otherwise grossly normal. A CT scan with contrast (Figures 1-3) performed in the emergency department was consistent with ileocecal intussusception of less than 7 cm and displayed a small bowel obstruction, target sign, and crescent sign.

**Figure 4:**
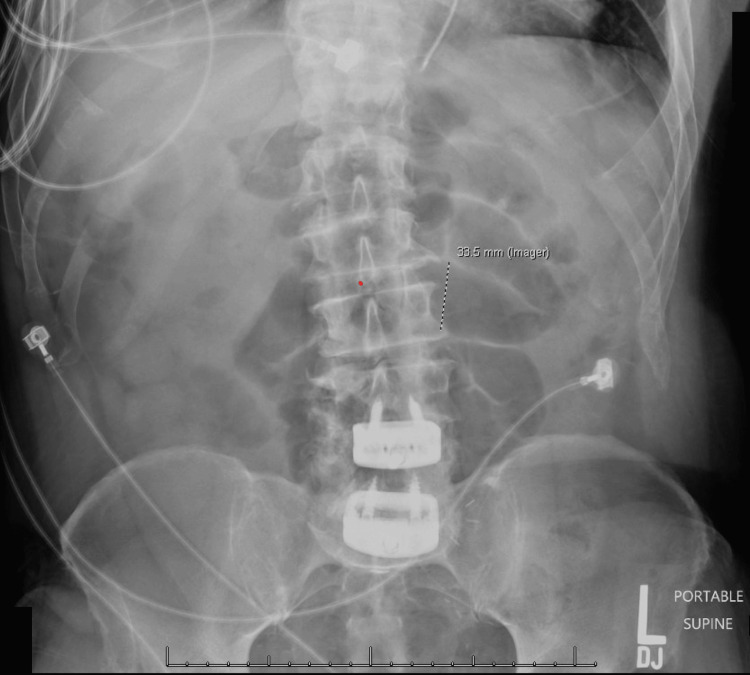
X-ray showing small bowel obstruction

The patient was subsequently admitted for symptomatic management and conservative treatment was employed. He was observed in the hospital for two days for analgesia and gastric decompression via a nasogastric tube. Despite these interventions, follow-up plain films (Figure 4) continued to indicate signs of obstruction including dilated, gas-filled, central enteric loops. The patient remained essentially asymptomatic.

The patient underwent an exploratory laparoscopy and subsequent right hemicolectomy with extracorporeal side-by-side anastomosis. A 7-cm section of intussusception of the ileum into the cecum was observed. Reducing the intussusception was not preferred due to friable tissue and the risk of perforation. Therefore, a right hemicolectomy was performed to resect the compromised bowel. Intraoperative examination did not reveal any visible mass or signs of malignancy that suggested a lead point. The mesentery and 15 lymph nodes were removed for pathologic examination due to suspected lymphovascular invasion. The patient had a normal postoperative recovery without significant complications. 

**Figure 5:**
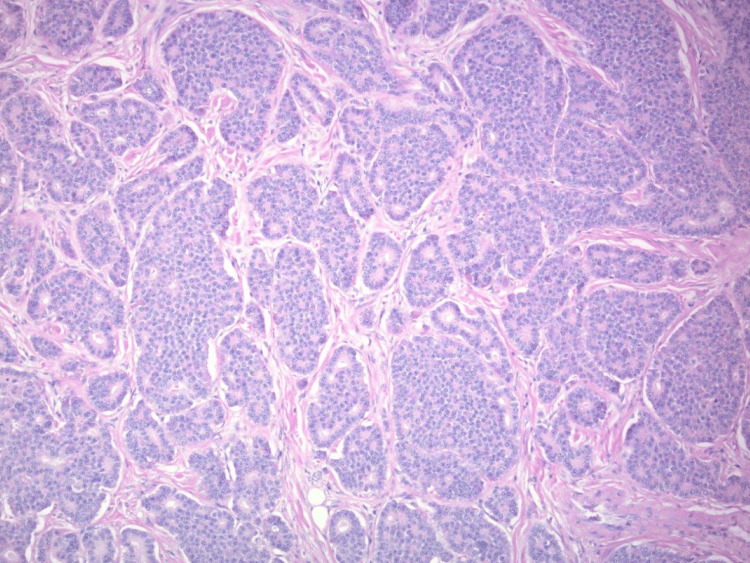
Histologic features of carcinoid tumor at high power

**Figure 6:**
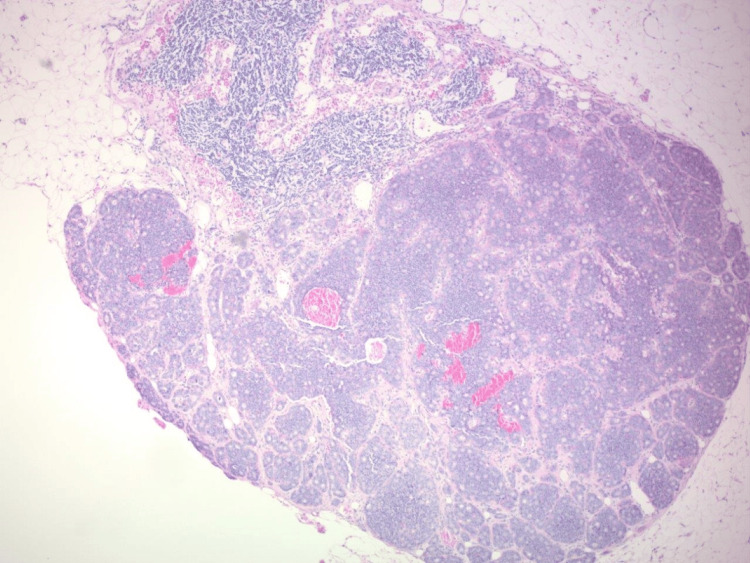
Low-power view of carcinoid tumor within identified lymph node metastasis

The final pathological evaluation identified a pT3n1m0 Grade 1 Neuroendocrine tumor of the colon originating in the ileum and spreading to the small intestine and colonic mesenteric nodes (Figure 5). Three of the 15 resected nodes were positive for metastases (Figure 6). After the hemicolectomy and resection, no further treatment was indicated. After a pathologist review of the resected tumor, it revealed a grade one well-differentiated carcinoid tumor. With no symptoms of carcinoid syndrome, it was determined that it was appropriate to move into surveillance with no further treatment.

## Discussion

Intussusception in adults is a seldom encountered yet significant contributor to bowel obstruction, boasting an incidence rate of approximately one in 1300 abdominal operations [13]. Notably, neoplasms emerge as the primary instigator in 69% of cases, with malignancies comprising a staggering 70% of these instances [6]. This distinctive case unfolds with intussusception secondary to a carcinoid tumor. Interestingly, the incidence of carcinoid tumors (all types) is 1.2-2.1/100,000 while the incidence of carcinoid syndrome is 0.5/100,000 [14]. What distinguishes this presentation is the absence of overt cancer symptoms prior to the patient seeking medical attention, underscoring the critical need for a heightened clinical suspicion when confronting unusual etiologies. 

Carcinoid tumors in the United States are rare neuroendocrine neoplasms that occur in an estimated 3.56 cases per 100,000 people [12]. Carcinoid tumors are the most common small bowel tumor accounting for 20-50% of all small bowel malignancies and are more frequently identified in the ileum [3]. In the context of carcinoid tumors causing ileocecal intussusception, the available literature reveals limited reported cases, emphasizing the rarity of this presentation in the medical literature. Notably, in a documented instance, a 53-year-old male presented with hematochezia accompanied by signs of ongoing obstruction, leading to the identification of ileocecal intussusception originating from a carcinoid tumor [11]. This patient was lost to follow-up but biomarkers did not suggest metastatic disease. Another reported case involved a 45-year-old male whose primary complaint was vague abdominal discomfort that progressively increased over several weeks [15]. In this instance, a CT scan revealed ileocecal intussusception, prompting surgical intervention. Subsequent ileocecal resection unveiled a small carcinoid tumor located at the terminal ileum.

According to recent guidelines from the American Joint Committee on Cancer (AJCC) and the National Comprehensive Cancer Network (NCCN), more than 12 lymph nodes should be removed in the case of right-sided stage II colon cancer without additional risk factors [16]. A study by Hwang et al. showed that survival outcomes of patients with a harvest of more than 25 nodes were not significantly different compared with those in whom less than 25 were removed [16]. These cases contribute valuable insights into the diverse ways carcinoid tumors can manifest and further underscore the need for heightened clinical suspicion when encountering ileocecal intussusception of unknown etiology.

Possible complications of intussusception often result from a delay in diagnosis and treatment due to a vague clinical picture and a vast differential diagnosis. Complications can include bowel perforation, bowel necrosis and ischemia, peritonitis, sepsis, and metastatic spread [13]. Given that the current patient presented without a fever, white count, peritoneal exam findings, or several clinical presentations, it was decided to start with a trial of conservative management. 

The postoperative surveillance plan for the current patient comprised routine imaging studies, including repeat CT scans every three to four months, chest CT for staging, and serial surveillance CTs in the first year, followed by annual scans over the next 10 years. To specifically evaluate for metastasis, a PET scan is scheduled approximately six months post-op. This comprehensive imaging approach should be complemented by regular colonoscopies under the supervision of the general surgeon.

According to the NCCN guidelines, monitoring lab values such as chromogranin A and 5-HIAA every three to six months may be indicated for detecting metastatic disease post-tumor removal [17]. However, it's noteworthy that the current patient did not undergo preoperative or postoperative chromogranin A or 5-HIAA testing, preventing the establishment of a baseline for subsequent surveillance. The decision to omit testing for these markers was partially influenced by the absence of signs and symptoms of serotonin syndrome in the patient. Additionally, a comprehensive imaging surveillance plan, which includes a PET scan with a radio-labeled somatostatin analog, was already in place alongside the recommended serial CT scans. While these markers are commonly used to monitor for metastasis or recurrence, their absence in our case limits their utility in the ongoing surveillance strategy. 

While not directly applicable to the current case presentation, consideration for additional therapeutic interventions includes the evaluation of octreotide and somatostatin treatments, particularly in the context of carcinoid syndrome. Octreotide treatment has shown significant promise, with over 70% of patients experiencing symptomatic improvement in cases of the syndrome [14]. Patients manifesting symptomatic carcinoid syndrome typically receive a long-acting release of octreotide at a dose of 20-30 mg intramuscularly every four weeks. Beyond symptom control, studies have revealed that octreotide can extend survival in patients with metastatic carcinoid tumors, increasing the average lifespan from 19 to 39 months [18].

This enhanced survival rate can be attributed to octreotide's efficacy in managing potential life-threatening consequences associated with carcinoid crisis, such as flushing, diarrhea, and valvular heart disease. Additionally, the observed increase in life expectancy may be linked to the potential antiproliferative effects of octreotide on carcinoid tumors. Octreotide is well-tolerated, has minimal side effects, and has even demonstrated a 10% reduction in tumor burden [14].

Furthermore, in cases involving liver metastasis, the consideration of a liver biopsy gains significance, especially given its heightened occurrence in the context of carcinoid syndrome [11]. This comprehensive approach aims not only to alleviate symptoms but also to address the underlying factors contributing to carcinoid metastasis, offering a more nuanced and effective management strategy.

The prognosis for patients with carcinoid or neuroendocrine tumors of the ileum is highly variable; 58-64% of patients with carcinoid tumors of the distal small bowel had metastatic spread at the time of diagnosis [3]. The current patient had the additional complication of intussusception. However, this was a silver lining as despite the elusive nature of neuroendocrine tumors and the likelihood of metastasis, the intussusception allowed for the early discovery of resection and early surveillance of a potentially life-threatening disease. 

## Conclusions

This case of adult intussusception caused by a carcinoid tumor highlights the necessity of considering uncommon etiologies in adult patients presenting with this condition. The initial conservative approach, guided by the patient's clinical presentation, lack of acute signs, and absence of significant risk factors reflects the dynamic nature of intussusception which may resolve spontaneously. This case underscores the challenges in diagnosing adult intussusception and the essential role of imaging, particularly CT scans, in achieving a definitive diagnosis. The absence of typical carcinoid syndrome symptoms in our patient emphasizes the diverse and atypical presentations of carcinoid tumors, necessitating a comprehensive diagnostic approach. The postoperative management and surveillance plan outlined in this case demonstrates the intricate balance required for long-term patient care, emphasizing the need for ongoing research to deepen our understanding of this rare condition and refine treatment strategies.
